# Peri-Implant Gingival Undifferentiated SWI/SNF Complex-Deficient Tumor with Molecularly Confirmed Biallelic SMARCA4 Inactivation: Diagnostic Pitfalls and Genomic Characterization

**DOI:** 10.3390/diagnostics16111732

**Published:** 2026-06-04

**Authors:** Haim Ohayon, Ahmad Hija, Amir Bilder, Tal Capucha, Sharon Akrish, Amir Wolff, Omri Emodi

**Affiliations:** 1 Department of Oral and Maxillofacial Surgery, Rambam Health Care Campus, Haifa 3109601, Israel; 2 The Ruth & Bruce Rappaport Faculty of Medicine, Technion–Israel Institute of Technology, Haifa 3109601, Israel

**Keywords:** SMARCA4, BRG1, SWI/SNF complex, undifferentiated tumor, peri-implant gingiva, dental implants, next-generation sequencing, MTAP, chromatin remodeling, head and neck oncology, case report

## Abstract

**Background and Clinical Significance**: SWI/SNF chromatin remodeling complex-deficient malignancies constitute an aggressive group of undifferentiated tumors defined by inactivation of core subunits including SMARCA4 (BRG1) or SMARCB1 (INI1). In the head and neck, these tumors predominate in the sinonasal tract; oral cavity presentations are exceedingly rare, with reported cases predominantly representing metastatic disease. Peri-implant gingival masses in clinical practice are overwhelmingly reactive, but their occasional malignant nature mandates timely biopsy and thorough pathologic workup. We report the first comprehensively molecularly characterized case of a peri-implant gingival SWI/SNF complex-deficient tumor with confirmed biallelic SMARCA4 inactivation. **Case Presentation**: A 75-year-old man presented with a one-week history of a rapidly enlarging exophytic erythematous peri-implant gingival mass in the right posterior mandible (region 44–47). Incisional biopsy demonstrated an undifferentiated high-grade tumor with epithelioid, plasmablastoid, and focally rhabdoid morphology with necrosis. Immunohistochemistry showed complete loss of BRG1 (SMARCA4) with retained INI1 (SMARCB1), EMA positivity, Ki-67 of approximately 100%, and negativity across all lineage-specific markers (hematolymphoid, epithelial, melanocytic, endothelial, squamous). Comprehensive next-generation sequencing (Oncomine Comprehensive Assay Plus) confirmed biallelic SMARCA4 inactivation via a truncating nonsense mutation (p.Trp1346Ter; VAF 73.85%) combined with copy number loss, establishing the molecular mechanism underlying BRG1 protein loss. Co-occurring alterations included homozygous CDKN2A/CDKN2B deletion, MTAP loss (9p21.3), clonal TP53 and KEAP1 mutations, and intermediate–high tumor mutational burden (13.3 mutations/Mb) with microsatellite stability. The patient initiated carboplatin–paclitaxel and achieved a partial response at one month with further shrinkage by four months. This case illustrates a rare oral cavity manifestation of SWI/SNF complex deficiency arising in a peri-implant location, with a diagnostic workup that required integration of immunohistochemistry and molecular profiling for definitive characterization. The MTAP deletion co-occurring with homozygous CDKN2A/B loss identifies a potentially actionable synthetic lethal vulnerability to MAT2A and PRMT5 inhibitors currently under clinical investigation. An occult primary site could not be fully excluded due to absence of a dedicated staging workup. **Conclusions**: Rapidly enlarging peri-implant gingival masses should prompt timely biopsy and SWI/SNF marker testing when histology is high-grade and lineage-ambiguous. NGS-based molecular profiling confirms diagnosis, elucidates mechanism, and reveals actionable targets in this rare tumor class.

## 1. Introduction

The SWI/SNF (SWItch/Sucrose Non-Fermentable) chromatin remodeling complex is a multisubunit ATP-dependent macromolecular assembly responsible for nucleosome repositioning, gene transcription regulation, and maintenance of cellular differentiation [1,2]. Encoded by more than 20 subunit genes, the SWI/SNF complex functions as a critical tumor suppressor across virtually all tissue types; collectively, SWI/SNF subunit mutations are estimated to occur in approximately 20% of all human cancers, rendering it among the most frequently mutated complexes in the human cancer genome [2]. Inactivation of individual subunits—most notably SMARCB1 (INI1), SMARCA4 (BRG1), SMARCA2 (BRM), ARID1A, and ARID1B—defines distinct tumor entities that share the property of aggressive, often undifferentiated behavior and poor response to standard cytotoxic regimens [1,2].

SMARCA4 encodes BRG1, the primary ATPase catalytic subunit of the canonical (cBAF) and PBAF SWI/SNF subcomplexes [1]. Biallelic inactivation of SMARCA4, typically through point mutation combined with loss of heterozygosity, results in complete loss of BRG1 protein expression detectable by immunohistochemistry—a finding now incorporated into the diagnostic criteria for several distinct tumor entities [3,4,5,6]. SMARCA4-deficient malignancies were first systematically described in the thoracic cavity, where SMARCA4-deficient thoracic sarcoma/undifferentiated carcinoma represents an aggressive entity predominantly affecting young smokers [7,8]. Subsequent recognition of SMARCA4-deficient uterine tumors and soft tissue sarcomas further established the cross-tissue relevance of this oncogenic mechanism [9,10].

In the head and neck, SWI/SNF complex deficiency has emerged as a defining axis for a heterogeneous group of aggressive undifferentiated malignancies [3]. The sinonasal tract is the most established head and neck site, where both SMARCB1-deficient and SMARCA4-deficient carcinomas have been well characterized in dedicated series and reviews [3,4,5,11,12,13,14,15]. SMARCB1(INI1)-deficient sinonasal carcinoma and SMARCA4/BRG1-deficient sinonasal carcinoma represent the two principal entities, with overlapping morphologic features including high-grade undifferentiated histology, epithelioid or plasmablastoid growth pattern, and frequent rhabdoid differentiation [3,4,11,12,13,15]. Both entities are associated with aggressive clinical behavior, high recurrence rates, and poor overall prognosis [4,11,12]. The WHO 5th edition classification of head and neck tumours (2022) formally incorporates SWI/SNF-deficient entities within the sinonasal carcinoma category, reflecting the consolidated diagnostic and prognostic importance of these alterations [6]. This classification framework also contextualizes SWI/SNF-deficient sinonasal carcinomas within a broader spectrum of molecularly defined undifferentiated sinonasal and head and neck tumors that share morphologic overlap but are distinguished by molecular features—including SMARCA4-deficient thoracic-type undifferentiated carcinoma (TCS), IDH2-mutant sinonasal undifferentiated carcinoma, and NUT carcinoma—a distinction directly relevant to the differential diagnosis of any high-grade undifferentiated tumor arising outside the sinonasal tract [6,16,17].

Outside the sinonasal tract, SMARCA4-deficient tumors have been documented at other head and neck sites. Primary oropharyngeal SMARCA4-deficient carcinoma has been described [18], and a case of metastatic SMARCA4-deficient undifferentiated carcinoma involving the oral cavity and mandible has been reported, with the explicit recommendation to evaluate for a distant primary when such a lesion is encountered in this location [19]. Oral cavity involvement by SMARCA4-deficient tumors warrants conceptual clarity across three anatomic categories: (1) primary sinonasal or extra-sinonasal head and neck tumors, including oropharyngeal cases such as that described by Pasricha et al. [18]; (2) oral metastatic SMARCA4-deficient carcinoma originating from a distant primary, as documented by Kim et al. [19]; and (3) tumors arising primarily within the oral cavity or gingiva in the absence of an identifiable alternative primary site. Lesions in the third category remain exceptionally rare in the indexed literature, with no prior report providing comprehensive NGS-based molecular characterization. The expanding use of comprehensive next-generation sequencing (NGS) in clinical oncology has begun to reveal the broader co-mutational landscape of SWI/SNF-deficient tumors beyond the defining SMARCA4 or SMARCB1 inactivation, including co-occurring alterations in CDKN2A, CDKN2B, MTAP, TP53, and KEAP1 with potentially actionable therapeutic implications [20,21,22,23].

Peri-implant gingival lesions are encountered with increasing frequency in oral and maxillofacial surgical practice, driven by the growing prevalence of osseointegrated dental implants worldwide [24]. The vast majority of peri-implant soft tissue lesions are reactive or inflammatory in nature, including peri-implant mucositis, pyogenic granuloma, and fibrous hyperplasia; malignancies arising in direct association with dental implants are distinctly uncommon [25,26]. However, reports of squamous cell carcinoma and, rarely, other malignancies presenting as peri-implant soft-tissue masses have established the critical importance of histopathologic evaluation when clinical features are atypical or when growth is rapid [25,26]. The clinical mimicry of malignancy by reactive peri-implant conditions constitutes a recognized diagnostic pitfall that can delay diagnosis and adversely affect prognosis. In this context, the pretest probability of malignancy is low, and squamous cell carcinoma—when suspected—typically presents with surface mucosal irregularity, induration, keratinization, and a history of tobacco or alcohol exposure. The present case, characterized by rapid onset, non-keratinizing exophytic growth, absence of surface ulceration of obvious epithelial origin, negativity for all squamous markers (p40, p63), and occurrence in a non-smoking patient without prior mucosal disease, reduced the a priori likelihood of conventional SCC and prompted broader diagnostic consideration that ultimately led to SWI/SNF immunohistochemistry.

We report a peri-implant mandibular gingival undifferentiated SWI/SNF complex-deficient tumor confirmed by immunohistochemistry and, for the first time at this oral anatomic site, comprehensive NGS molecular profiling. We describe the diagnostic approach, the molecular landscape including potentially actionable alterations, and review the relevant literature to contextualize this exceptionally rare presentation.

## 2. Case Report

**Figure 1:**
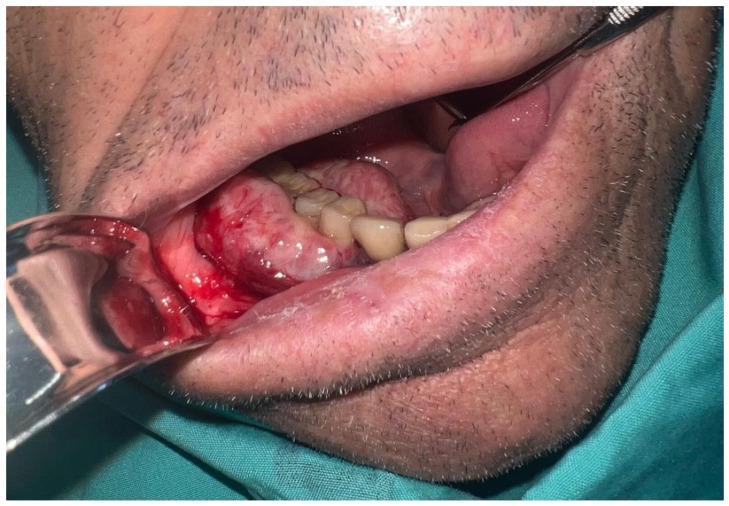
Clinical photograph demonstrating an exophytic erythematous peri-implant gingival mass in the right posterior mandible (region 44–47) at presentation. The lesion involves both buccal and lingual gingiva in the vicinity of osseointegrated implants, without purulence or fluctuance.

A 75-year-old man presented in September 2025 with approximately one week of progressive swelling in the right mandibular posterior gingiva. His medical history was significant for non-Hodgkin lymphoma (in remission, under active surveillance), obstructive sleep apnea, prior cerebrovascular accident, and type 2 diabetes mellitus. He was a non-smoker with no prior history of head and neck malignancy. Dental history was notable for osseointegrated implants placed in the right posterior mandible approximately 10 years prior, with no documented peri-implant complaints until the preceding year. The patient had attended a dentist in the period immediately before presentation and had been prescribed a chlorhexidine mouthwash prior to referral to our department. General intraoral examination revealed no additional mucosal lesions or cervical lymphadenopathy on clinical palpation. At the site of interest, examination demonstrated a red, exophytic peri-implant gingival mass involving both buccal and lingual gingiva around osseointegrated implants in the right posterior mandible, region 44–47 (Figure 1). The mass measured approximately 2–3 cm in its greatest dimension, was non-fluctuant and non-suppurative, with no clinical evidence of purulence or fistula formation. Panoramic radiography did not demonstrate a primary bony destructive process, suggesting soft-tissue confinement at presentation.

**Figure 2:**
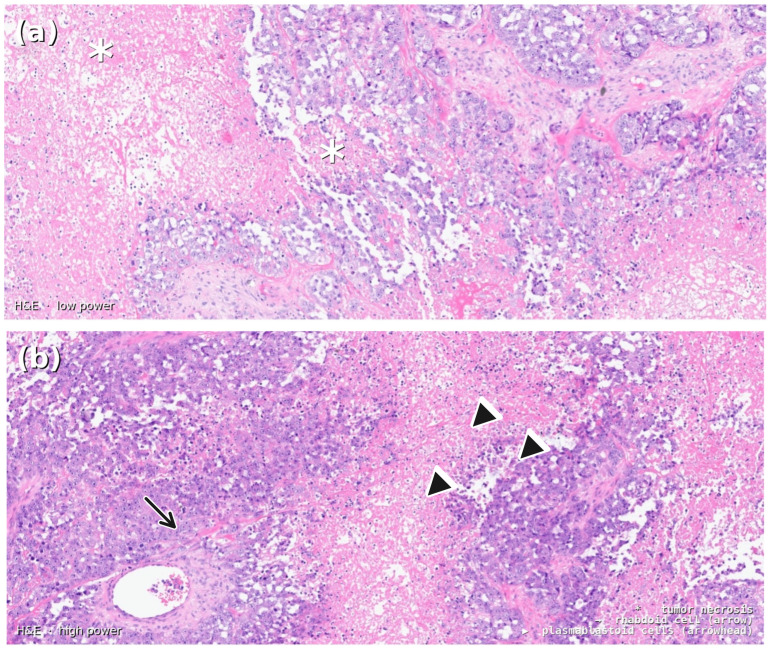
Histopathology. (**a**) Low-power hematoxylin and eosin (H&E) section showing a diffuse, undifferentiated high-grade neoplasm composed of sheets of epithelioid and plasmablastoid cells, with geographic tumor necrosis (asterisk) and brisk mitotic activity. (**b**) High-power H&E showing plasmablastoid morphology, with moderate to abundant pale eosinophilic cytoplasm and vesicular nuclei bearing prominent nucleoli (arrowheads), alongside a rhabdoid cell (arrow).

**Figure 3:**
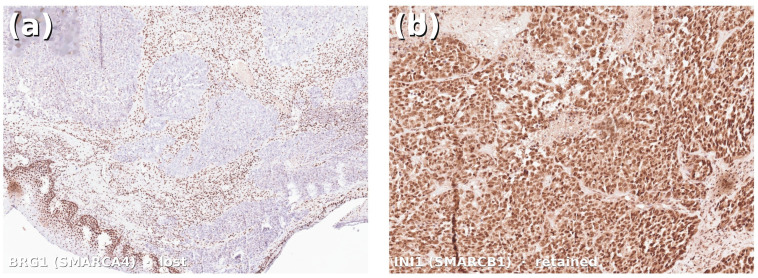
Immunohistochemistry. (**a**) BRG1 (SMARCA4) immunohistochemistry demonstrating complete loss of nuclear expression in tumor cells (pale nuclei) with retained brown nuclear positivity in internal stromal controls, confirming true protein loss. (**b**) INI1 (SMARCB1) immunohistochemistry showing diffuse strong retained nuclear expression throughout all tumor cells, confirming that SWI/SNF deficiency is selective to SMARCA4.

Given the clinical differential diagnosis—which encompassed reactive peri-implant lesion, squamous cell carcinoma, lymphoma, and other soft-tissue malignancy—an incisional biopsy was performed on 12 October 2025. Histopathologic evaluation demonstrated an undifferentiated high-grade neoplasm with epithelioid and plasmablastoid morphology, focally rhabdoid differentiation, prominent tumor necrosis, and a proliferation index approaching 100% on Ki-67 immunohistochemistry (Figure 2 and Figure 3). The immunohistochemical workup—undertaken systematically to exclude relevant diagnostic entities including high-grade carcinoma, lymphoma, melanoma, and vascular malignancy—identified complete loss of BRG1 (SMARCA4) protein expression with retained INI1 (SMARCB1), establishing a diagnosis of undifferentiated SWI/SNF complex-deficient tumor (detailed in Section 3). A whole-body FDG PET/CT, available from prior lymphoma surveillance, identified no hypermetabolic lesion suspicious for an alternative primary site. The case was subsequently reviewed at a multidisciplinary tumor board, at which no clinically apparent alternative primary was identified.

**Figure 4:**
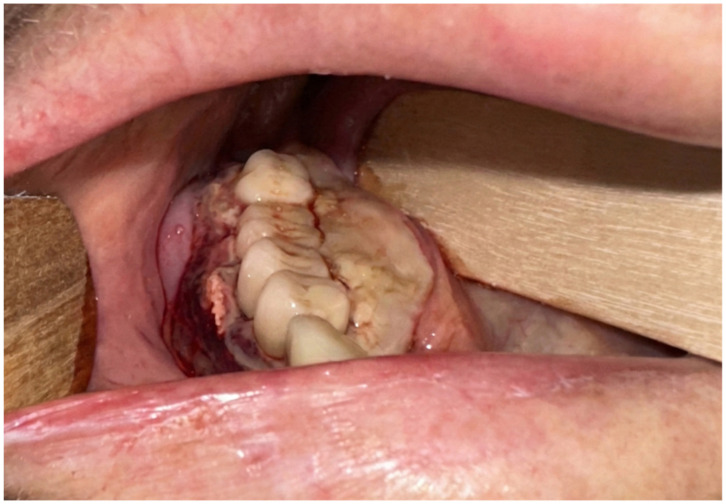
Clinical photographs following initiation of carboplatin–paclitaxel chemotherapy (23 November 2025) demonstrating partial response and further tumor shrinkage at approximately four months, with persistent residual disease at the time of manuscript preparation.

In light of the confirmed high-grade undifferentiated diagnosis, the aggressive histologic and molecular profile, and the absence of a surgically resectable primary site amenable to curative-intent surgery, the tumor board consensus was to proceed with systemic therapy. The patient was referred to medical oncology and initiated carboplatin and paclitaxel on 23 November 2025. Clinical and photographic assessment demonstrated a partial response at approximately one month, with further tumor shrinkage documented at approximately four months; residual disease was present at the time of manuscript preparation (Figure 4).

Written informed consent for publication of this case report and accompanying images was obtained from the patient.

## 3. Pathologic and Molecular Findings

### 3.1. Histopathology

Incisional biopsy demonstrated an undifferentiated high-grade neoplasm with epithelioid and plasmablastoid cell morphology, focally rhabdoid differentiation, and prominent tumor necrosis (Figure 2). Cells showed moderate to abundant pale eosinophilic cytoplasm, vesicular nuclei with prominent nucleoli, and high mitotic activity consistent with a proliferation index of approximately 100% on Ki-67 immunohistochemistry. No glandular, squamous, or melanocytic differentiation was identified on routine sections.

The complete immunohistochemical profile is summarized in Table 1. The principal diagnostic finding was complete loss of BRG1 (SMARCA4) protein expression, confirmed with appropriate positive stromal internal control, in the context of retained INI1 (SMARCB1). This pattern—selective BRG1 loss with INI1 retention—is the immunohistochemical signature of SMARCA4-deficient SWI/SNF complex-deficient tumors [3,4,5,11]. EMA was positive and CD138 showed weak expression; cyclin D1 and c-MYC were positive. Comprehensive lineage exclusion demonstrated negativity across all tested hematolymphoid markers (CD45, CD20, CD19, PAX5, CD3, CD5, CD10, CD38, MUM1, BCL6, kappa and lambda light chains), all tested epithelial keratins (CK7, CK20), squamous markers (p40, p63), melanocytic markers (S100, SOX10), endothelial markers (CD31, CD34, ERG), and BerEP4.

A broad-spectrum pan-cytokeratin stain (AE1/AE3) was not included in the initial immunohistochemical panel. The negative CK7, CK20, p40, and p63 results, taken together with the molecular profile (see Section 3.2), support the conservative designation ‘undifferentiated SWI/SNF complex-deficient tumor,’ consistent with WHO 2022 guidance for cases where pan-cytokeratin confirmation is unavailable [6].

Focal LMP1 immunoreactivity was noted. The staining pattern was assessed as non-specific based on a diffuse cytoplasmic rather than membranous distribution; EBV-ISH (EBER) was unequivocally negative, excluding EBV-associated pathology.

### 3.2. Molecular Profile (NGS)

Comprehensive genomic profiling was performed using the Oncomine Comprehensive Assay Plus panel (next-generation sequencing; Rambam Health Care Campus Molecular Pathology Laboratory; assay covering point mutations, fusions, and copy number variations; report dated January 2026). Tumor cellularity was 80%. The full molecular profile is presented in Table 2.

The principal finding was biallelic SMARCA4 inactivation. A truncating nonsense mutation, SMARCA4 p.Trp1346Ter (c.4038G>A; W1346*), was identified at a variant allele frequency (VAF) of 73.85% (589 reads), consistent with a clonal, pathogenic event. SMARCA4 additionally demonstrated copy number loss (1.2 copies at the 19p13.2 locus). The co-occurrence of a truncating loss-of-function mutation on one allele with reduced copy number of the locus constitutes a two-hit biallelic inactivation mechanism—the established molecular basis for complete BRG1 protein loss as detected by immunohistochemistry [1,2,8,20].

Co-occurring somatic alterations of clinical significance included: (1) Homozygous deletion of CDKN2A (0 copies) and CDKN2B (0 copies) at the 9p21.3 locus, resulting in complete abrogation of p16/INK4A, p14/ARF, and p15/INK4B tumor suppressor function. (2) MTAP copy number loss (0.5 copies) at the same 9p21.3 locus, co-deleted due to genomic proximity. (3) Clonal TP53 p.Arg213Leu mutation (VAF 65.92%). (4) Clonal KEAP1 p.Ala344LeufsTer56 frameshift mutation (VAF 71.80%), a recurrent alteration activating the NRF2 oxidative stress pathway and associated with chemoresistance in multiple tumor types. (5) Subclonal CTNNB1 p.Ser37Phe (VAF 23.49%), an activating beta-catenin mutation at a phosphorylation site relevant to Wnt pathway regulation. An SDHB p.Arg230Cys variant (VAF 54.05%) was classified as a variant of uncertain significance; SDHB protein expression was retained on IHC, arguing against biallelic protein inactivation, and germline genetic counseling was recommended given the allele frequency and SDH-related syndrome associations.

Tumor mutational burden (TMB) was 13.3 mutations/Mb. Microsatellite status was microsatellite-stable (MSS; score 3.24). No gene fusions were detected.

## 4. Discussion

### 4.1. SWI/SNF Complex-Deficient Tumors: Biological Context and Expanding Spectrum

The SWI/SNF complex functions as a master regulator of chromatin accessibility and transcriptional programming, with essential roles in cell fate specification, differentiation, and proliferation control [1,2]. The tumor-suppressive function of the complex is underscored by the frequency and breadth of SWI/SNF subunit mutations across cancer types; inactivation of individual subunits deregulates thousands of target genes, promoting dedifferentiation, immune evasion, and resistance to apoptosis [1,2,23]. SMARCA4/BRG1 inactivation is the most common single-subunit alteration in SWI/SNF-deficient malignancies outside the central nervous system, with well-characterized tumor entities defined by this alteration in the thorax, gynecologic tract, and head and neck [6,7,8,9].

In the thoracic cavity, SMARCA4-deficient undifferentiated carcinoma/thoracic sarcoma was among the earliest described entities in this class [7,8]. These tumors predominantly arise in male smokers, typically present with advanced disease, and demonstrate highly aggressive behavior with poor prognosis despite multimodal therapy [8,22]. SMARCA4-deficient uterine tumors, including dedifferentiated endometrial carcinoma and undifferentiated uterine sarcoma, represent another well-characterized entity with distinct molecular and clinicopathologic features [9]. The recognition that SMARCA4 inactivation can drive aggressive undifferentiated neoplasia across diverse tissue types has important diagnostic implications, highlighting the need for BRG1 immunohistochemistry as part of the workup of any high-grade undifferentiated tumor when initial lineage markers are non-contributory [3,4,5,6].

### 4.2. Head and Neck SWI/SNF-Deficient Tumors and the Sinonasal Paradigm

The sinonasal tract is the predominant head and neck site for both SMARCB1- and SMARCA4-deficient tumors [3,4,5,13,14]. SMARCB1(INI1)-deficient sinonasal carcinoma, first described by Agaimy et al. in 2017, is characterized by basaloid or plasmacytoid morphology, frequent rhabdoid differentiation, INI1 loss, and an exceptionally poor prognosis [13]. The subsequent description of SMARCA4/BRG1-deficient sinonasal carcinoma added a second major entity to this spectrum, with overlapping histologic features but the distinctive immunohistochemical signature of BRG1 loss with INI1 retention [5,12,15]. Larger series have confirmed consistent morphologic features—epithelioid or plasmablastoid growth, necrosis, Ki-67 approaching 100%, and aggressive clinical behavior—as well as the practical utility of incorporating BRG1 and INI1 immunohistochemistry into the diagnostic algorithm for high-grade undifferentiated sinonasal tumors [4,5,11,12].

The WHO 5th edition classification of head and neck tumours (2022) formally recognizes SMARCB1- and SMARCA4-deficient sinonasal carcinomas as distinct entities within the expanded classification of sinonasal malignancies [6]. Beyond the sinonasal tract, primary SMARCA4-deficient carcinoma has been described in the oropharynx [18], and the present case adds to the anatomic diversity of primary head and neck sites where SWI/SNF complex deficiency may manifest. The clinical and pathologic spectrum continues to expand with increasing awareness and broader application of SWI/SNF immunohistochemistry.

### 4.3. Oral Cavity Involvement and the Peri-Implant Presentation

Oral cavity involvement by SMARCA4-deficient tumors is distinctly rare in the indexed literature. Kim et al. (2025) described a case of metastatic SMARCA4-deficient undifferentiated carcinoma presenting in the oral cavity and mandible, with immunohistochemical confirmation of BRG1 loss and the explicit recommendation to evaluate for a distant primary when such a lesion is encountered in this location [19]. The present case differs in several critical respects: the tumor presented in direct association with dental implants arising from peri-implant gingival soft tissue; multidisciplinary tumor board review did not identify a clinically apparent alternative primary site; and comprehensive NGS profiling was performed, revealing a complex co-mutational landscape not previously described for a SWI/SNF-deficient tumor at this anatomic site. A dedicated staging workup—including contrast-enhanced CT of the neck, chest, abdomen, and pelvis—was not performed as a site-specific investigation for the oral lesion; the available systemic imaging consisted of a whole-body FDG PET/CT obtained in the context of lymphoma surveillance. FDG PET/CT carries recognized limitations for small or superficial mucosal primaries [27], and accordingly an occult primary site cannot be fully excluded. This limitation has direct implications that must be addressed explicitly. First, regarding the novelty claim: if this lesion represents an oral metastasis from an occult primary, it would parallel the Kim et al. case [19] rather than extending the anatomic spectrum of primary oral SWI/SNF-deficient tumors; the molecular co-mutational profile would remain of independent interest, but the site novelty claim would require qualification. Second, regarding site assignment: the designation as a primary oral presentation is a working clinical diagnosis based on multidisciplinary board review and available imaging, not a confirmed anatomic classification. Third, regarding therapeutic decision-making: systemic chemotherapy is appropriate for high-grade undifferentiated disease regardless of whether this is a primary or metastatic oral lesion, as curative surgical resection was not feasible in either scenario. We present the available evidence transparently and acknowledge that the primary designation cannot be confirmed without definitive staging; this reflects a real and irreducible limitation of the case rather than an analytical oversight.

The peri-implant presentation adds a clinically important dimension to this case. Dental implant failure and peri-implant soft tissue abnormalities are a common clinical scenario, and the vast majority represent reactive or infectious processes [24,25]. Malignancies arising at or around dental implant sites are infrequent but documented, with squamous cell carcinoma representing the most commonly reported implant-associated malignancy [25,26]. Systematic reviews of implant-associated malignancy have highlighted the importance of biopsy for rapidly growing or morphologically atypical peri-implant lesions and the risks of diagnostic delay when such lesions are attributed reflexively to peri-implantitis [26]. The present case reinforces this principle: a one-week history of rapid growth, erythema, and non-suppurative character in the context of pre-existing implants should prompt expedient biopsy rather than empirical treatment of presumed reactive disease.

### 4.4. Diagnostic Reasoning and Differential Diagnosis

The differential diagnosis for an undifferentiated high-grade oral soft-tissue tumor with plasmablastoid and rhabdoid morphology is broad and requires systematic exclusion of clinically important entities [3,11,19]. Poorly differentiated squamous cell carcinoma is the most common high-grade oral malignancy and should be the initial consideration; negativity for p40 and p63, along with absent keratinization, argues against this diagnosis in the present case. NUT carcinoma, characterized by NUT (NUTM1) rearrangements, shares morphologic overlap with SWI/SNF-deficient tumors and can also arise in the head and neck; NUT immunohistochemistry, which was not performed here but would be informative, is recommended in the workup of undifferentiated head and neck carcinomas [16,17].

Plasmablastic lymphoma represents a particularly important differential consideration given the plasmablastoid morphology and weak CD138 expression in this case. Plasmablastic lymphoma is an aggressive large B-cell lymphoma typically arising in immunocompromised patients, with a predilection for the oral cavity and jaw [28,29,30,31]. The absence of a definitive plasma cell immunophenotype—including negative CD38, MUM1, kappa and lambda light chains, and CD79a—alongside broad hematolymphoid marker negativity and unequivocally negative EBER in situ hybridization, effectively excludes plasmablastic lymphoma in typical diagnostic settings [28,29,30]. Metastasis from an extragingival SMARCA4-deficient tumor remains a recognized possibility, as highlighted by the Kim et al. case [19], and the absence of a dedicated systemic staging workup prevents definitive exclusion of this scenario in the present report. A further consideration merits explicit discussion: the immunophenotype in this case diverges from the profile typically reported in published SMARCA4-deficient sinonasal carcinoma series, which have generally demonstrated at least focal cytokeratin expression on broad-spectrum staining (AE1/AE3), with variable reactivity for neuroendocrine markers including synaptophysin and chromogranin [3,4,5]. In the present case, AE1/AE3 was not performed; CK7 and CK20 were negative; and synaptophysin and INSM1 were not included in the initial panel. This immunophenotypic profile makes direct equating of this tumor with the sinonasal SMARCA4-deficient carcinoma entity—as formally defined in the established series—diagnostically challenging. This uncertainty is precisely the rationale for the conservative designation of ‘undifferentiated SWI/SNF complex-deficient tumor’ rather than ‘SMARCA4-deficient carcinoma,’ consistent with WHO 2022 guidance for cases where pan-cytokeratin confirmation is unavailable [6]. The molecular confirmation of biallelic SMARCA4 inactivation establishes the SWI/SNF-deficient identity irrespective of this immunophenotypic gap, and future workup including AE1/AE3, synaptophysin, and INSM1 is recommended in analogous cases to enable more precise entity-level classification.

### 4.5. Molecular Basis of SMARCA4 Inactivation and Implications

The NGS confirmation of biallelic SMARCA4 inactivation in this case—through a truncating W1346* mutation at VAF 73.85% combined with copy number loss—provides the mechanistic explanation for complete BRG1 protein loss on immunohistochemistry. The high allele frequency of the truncating mutation indicates clonal dominance and a pathogenic driver role. This molecular validation is clinically meaningful: it resolves any interpretive ambiguity that can arise from IHC alone, particularly when tissue fixation conditions or antibody performance might be suboptimal, and it definitively establishes the SWI/SNF-deficient molecular identity of the tumor.

The co-occurring genomic alterations identified in this case are not incidental findings. Homozygous CDKN2A/CDKN2B deletion at 9p21.3 is a recognized aggressive co-driver in both SWI/SNF-deficient tumors and head and neck malignancies broadly [23]. Complete loss of p16/INK4A and p14/ARF through CDKN2A deletion abrogates both the RB pathway (via p16) and the MDM2-p53 axis (via p14/ARF), creating compounded cell-cycle deregulation. In a tumor already carrying clonal TP53 mutation, the additive effect of p14/ARF loss potentially further compromises p53-mediated apoptosis. The KEAP1 frameshift mutation at high VAF is known to activate the NRF2 oxidative stress pathway, a mechanism experimentally associated with platinum resistance in preclinical systems and suggested in some clinical datasets [23]; however, whether this translates to clinically meaningful resistance in the present case is speculative and cannot be inferred from a single patient without functional validation. Similarly, the compounded TP53/p14ARF pathway disruption and the subclonal CTNNB1 activation are noted as hypothesis-generating co-drivers based on published tumor biology, but their functional contribution to tumor behavior in this case remains unestablished. These observations are presented as potentially relevant contextual findings rather than mechanistic conclusions.

### 4.6. MTAP Deletion as a Therapeutically Actionable Target

Among the genomic findings, MTAP deletion merits particular emphasis for its therapeutic implications. MTAP (methylthioadenosine phosphorylase) is co-located with CDKN2A at chromosome 9p21.3 and is co-deleted in the majority of CDKN2A-homozygous tumors across cancer types [20,21]. MTAP encodes the rate-limiting enzyme of the methionine salvage pathway; its deletion causes accumulation of methylthioadenosine (MTA), which selectively inhibits PRMT5 (protein arginine methyltransferase 5) activity in tumor cells [20,21]. This creates a state of partial PRMT5 inhibition in MTAP-deleted tumor cells, rendering them hypersensitive to further PRMT5 inhibition or to MAT2A inhibition (which depletes the SAM substrate required for PRMT5 activity)—a synthetic lethal relationship exploitable therapeutically [20,21].

Both landmark studies by Mavrakis et al. and Kryukov et al. (2016) independently demonstrated that MTAP deletion confers selective vulnerability to PRMT5 and MAT2A inhibition in cancer cell lines and xenograft models [20,21]. Multiple Phase I/II clinical trials are now enrolling MTAP-deleted solid tumor patients, including trials of MAT2A inhibitors (IDE397, PRT-7732), PRMT5 inhibitors, tazemetostat-based combinations, and tucidinostat plus nivolumab and ipilimumab—agents directly relevant to this patient’s genomic profile [20,21,23]. The institutional NGS report identified 91 clinical trials relevant to this patient’s molecular profile, of which several specifically target MTAP-deleted tumors. This finding underscores the value of comprehensive NGS profiling in rare undifferentiated tumors: even in the absence of standard targeted therapy options, molecular profiling can identify enrollment eligibility for precision oncology trials.

### 4.7. Literature Review: SWI/SNF-Deficient Tumors—Reported Cases and Molecular Landscape

Since the initial descriptions of SMARCA4-deficient sinonasal carcinoma by Agaimy and Weichert (2017) [15] and subsequent series [4,5,12], the number of published cases has grown considerably, primarily within sinonasal anatomic boundaries. The expanded series by Agaimy et al. (2020) comprising 10 SMARCA4-deficient sinonasal carcinomas [12] and the concurrent growth of literature on SMARCB1-deficient entities [13] have collectively established the diagnostic and prognostic framework for SWI/SNF-deficient head and neck tumors within the WHO classification [6]. Published cases consistently demonstrate aggressive clinical behavior with high rates of local recurrence, nodal and distant metastasis, and limited durable response to conventional cytotoxic chemotherapy. Median survival across reported series is typically measured in months, reinforcing the need for novel therapeutic approaches.

Genomic profiling data for head and neck SWI/SNF-deficient tumors beyond the SMARCA4 or SMARCB1 inactivating event itself are limited in published literature. Available data suggest recurrent co-mutations in TP53, CDKN2A, and PIK3CA in SMARCB1-deficient sinonasal carcinomas [3,13], while comprehensive co-mutational profiling of SMARCA4-deficient head and neck tumors at the depth provided by clinical NGS panels has been reported in only isolated cases. The present case, demonstrating co-occurring homozygous CDKN2A/B deletion, MTAP loss, clonal TP53 and KEAP1 mutations, subclonal CTNNB1 activation, and intermediate–high TMB, provides among the most detailed molecular characterizations of a SWI/SNF-deficient tumor at an oral head and neck site currently available in the literature [23].

The SDHB p.Arg230Cys VUS identified at VAF 54.05% warrants specific discussion. SDHB mutations are associated with hereditary paraganglioma–pheochromocytoma spectrum disorders (Carney triad) and have been identified as somatic drivers in a subset of gastrointestinal stromal tumors [32]. The intermediate VAF in this case raises the possibility of a germline variant, which would have implications beyond the primary tumor diagnosis. Retained SDHB protein expression on IHC argues against biallelic inactivation at the protein level; however, germline testing was formally recommended given the allele frequency and the syndrome implications of pathogenic SDHB variants. This finding highlights the value of somatic NGS panels in identifying variants that warrant germline follow-up.

### 4.8. Limitations

This report carries limitations inherent to the single-case design. Generalizability and broader clinicopathologic conclusions are necessarily constrained by the absence of a comparator cohort; however, the extreme rarity of SWI/SNF-deficient tumors at this anatomic site renders multi-patient series impracticable in the near term, and single-case reports remain the appropriate and accepted vehicle for documenting novel presentations in this category [3,19]. A dedicated contrast-enhanced staging CT was not performed specifically for this oral lesion; the available systemic imaging was a surveillance FDG PET/CT for known lymphoma, which has recognized limitations for small mucosal primaries [27]. Accordingly, a distant occult primary cannot be definitively excluded. The immunohistochemical panel lacked broad-spectrum pan-cytokeratin (AE1/AE3) and NUT immunohistochemistry; these are noted in the text and appropriately mitigated by the comprehensive NGS data. Clinical follow-up was limited to approximately four months at the time of manuscript preparation, precluding long-term survival analysis. Functional validation of individual genomic alterations was not performed, and therapeutic implications for PRMT5/MAT2A inhibitors and other targeted agents remain theoretical, as the patient was not enrolled in a targeted trial. Germline confirmation of the SDHB variant of uncertain significance was recommended but not yet reported at the time of submission.

## 5. Conclusions

Rapidly enlarging peri-implant gingival masses should prompt expedient biopsy; high-grade undifferentiated histology warrants immediate SWI/SNF immunohistochemistry (BRG1 and INI1) as part of the diagnostic workup. In this case, NGS-confirmed biallelic SMARCA4 inactivation via a truncating mutation and copy number loss provided the molecular basis for BRG1 loss and definitively established tumor identity. The co-occurring genomic landscape—homozygous CDKN2A/B deletion, MTAP loss (a synthetic lethal target for MAT2A/PRMT5 inhibition), clonal TP53 and KEAP1 mutations, intermediate–high TMB, and a germline-relevant SDHB VUS—represents one of the more detailed molecular characterizations of a SWI/SNF-deficient tumor at an oral head and neck site currently available, acknowledging that similar NGS-level profiling has been reported in select extra-sinonasal head and neck cases. Comprehensive NGS profiling is strongly recommended in this rare tumor class: it confirms diagnosis, elucidates mechanism, and identifies potential clinical trial eligibility and hypothesis-generating genomic targets that warrant prospective investigation rather than immediate therapeutic inference.

## Figures and Tables

**Table 1:** Immunohistochemical profile.

Marker	Result
EMA	Positive
CD138	Positive (weak)
BCL1 (Cyclin D1)	Positive
c-MYC	Positive
Ki-67	~100%
INI1 (SMARCB1)	Retained (positive)
BRG1 (SMARCA4)	Lost (negative)—diagnostically decisive
LMP1	Focal; pattern assessed as non-specific (diffuse cytoplasmic, not membranous)
EBV-ISH (EBER)	Negative
HHV8	Negative
CD38/MUM1/CD79a/Light chains	All negative
PAX5/CD20/CD19/CD45	All negative
BCL2/BCL6/CD3/CD5/CD10/CD30	All negative
CK7/CK20/p40/p63	All negative
S100/SOX10	Negative
BerEP4	Negative
CD31/CD34/ERG	All negative

**Table 2:** Comprehensive molecular profile (Oncomine Comprehensive Assay Plus NGS; Rambam Molecular Pathology Laboratory; January 2026).

Category	Genomic Alteration	VAF/Copy Number	Notes
DNA Variants	SMARCA4 p.Trp1346Ter (W1346)	73.85%/589 reads	Truncating; pathogenic; two-hit with CNV loss
DNA Variants	TP53 p.Arg213Leu	65.92%/1030 reads	Clonal somatic
DNA Variants	KEAP1 p.Ala344LeufsTer56	71.80%/539 reads	Frameshift; NRF2 pathway activation
DNA Variants	CTNNB1 p.Ser37Phe	23.49%/1860 reads	Subclonal beta-catenin activation
DNA Variants	SDHB p.Arg230Cys	54.05%/2000 reads	VUS; IHC protein retained; germline counseling advised
Copy Number	SMARCA4	1.2 copies (LOSS)	Two-hit biallelic inactivation with W1346
Copy Number	CDKN2A	0 copies (HOMOZYGOUS)	9p21.3; complete p16/p14ARF loss
Copy Number	CDKN2B	0 copies (HOMOZYGOUS)	9p21.3; complete p15 loss
Copy Number	MTAP	0.5 copies (LOSS)	9p21.3; PRMT5/MAT2A synthetic lethal target
TMB/MSI	13.3 mut/Mb; MSS	—	No fusions detected

## Data Availability

All data relevant to this study are included in this article. Additional information is available from the corresponding author upon reasonable request, subject to applicable privacy regulations.
